# Complete Regression of Giant Inflammatory Polyp after Treatment with Adalimumab

**DOI:** 10.1155/2019/5347637

**Published:** 2019-03-07

**Authors:** Antoine Abou Rached, Leila El Masri, Mary Nakhoul

**Affiliations:** Lebanese University, School of Medicine, Lebanon

## Abstract

Giant inflammatory polyps (GIPs) are a rare complication of IBD resulting from chronic regenerative and healing processes leading to a polypoid formation on inflamed mucosa. We reported a case of GIP in a patient with long standing left-sided ulcerative colitis (UC); a well circumscribed sessile GIP was found during a colonoscopy for colorectal cancer screening on a normal colonic mucosa in the cecum. After a severe flare of the left-sided colitis and due to partial response to steroids patient was treated with adalimumab; new colonoscopy after 6 months shows complete disappearance of the GIP previously described. It is the first case report of GIP in normal macroscopic mucosa with complete disappearance after biologic treatment.

## 1. Case Presentation

**Figure 1:**
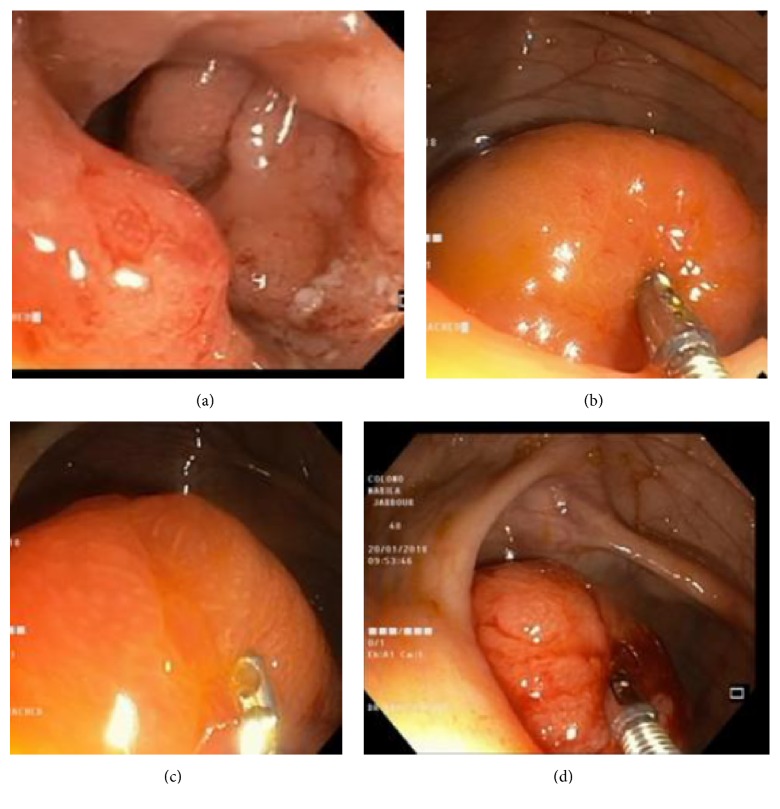
(a) Hyperaemia and diffuse superficial ulcerations of left colon. (b), (c), and (d) The giant pseudopolyp in the cecum with a normal colonic mucosa.

**Figure 2:**
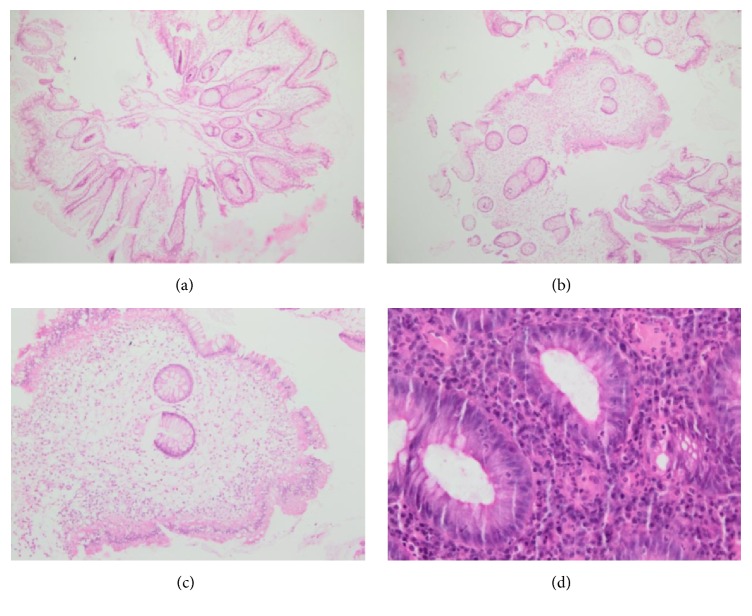
(a) Polypoid structure formed by disorganized and rarefied glands and crypts in an inflammatory and edematous lamina propria. (b)-(c) Inflammatory lamina propria and rarefied glands. (d) Cryptitis: exocytosis of polynuclear neutrophils in the glands.

A 48-year-old female patient was known to have left-sided ulcerative colitis (UC) since 2005 under treatment with oral (2 to 4 g/day) and rectal mesalamine with good clinical and biological response. She presented flare up in 2013 and 2015 with rapid clinical and biologic response to steroid therapy. She was in clinical and biologic remission since 2016. On January 2018, after 13 years of initial diagnosis and while she was clinically asymptomatic, a screening colonoscopy for colorectal cancer showed diffuse superficial ulcerations surrounded by an erythematous inflamed mucosa from the rectum to the splenic flexure (Figure 1(a)), a solitary sessile well circumscribed polyp in the cecum with normal adjacent mucosa, not amenable to endoscopic resection (Figures 1(b), 1(c), and 1(d)). Histologic examination revealed mucosal extension supported by submucosa consisting of a variable mixture of inflammatory tissue, which is histologically consistent with an inflammatory pseudopolyp (Figure 2)

**Figure 3:**
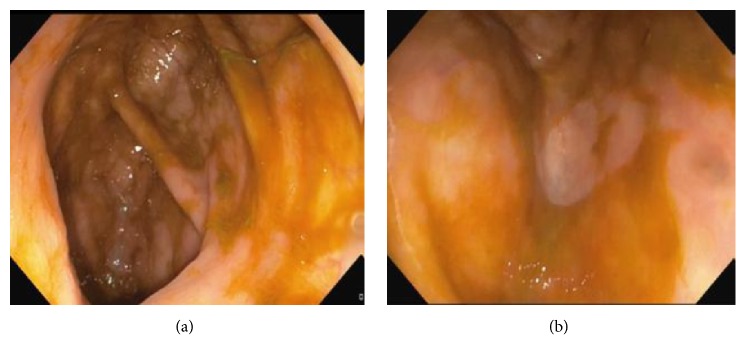
Complete resolution of cecal GIP after 8 months of adalimumab therapy.

**Figure 4:**
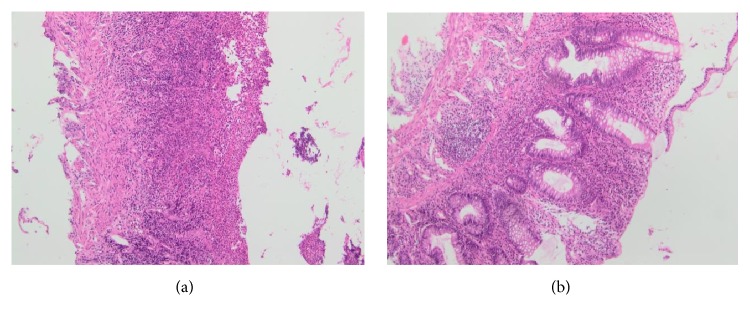
Desquamation and damage to the surface epithelium. Architectural distortion: duplication of crypts and irregular glands of varying sizes. Inflammatory infiltrate formed by neutrophils and plasmocytes in the lamina propria.

Two weeks later, she developed moderate flare up with bloody diarrhea and diffuse abdominal pain with no signs or symptoms of obstruction. Laboratory findings showed normal hemoglobin, albumin of 3.7 g/dl, normal liver function tests, and C-reactive protein of 25 (normal < 5). Infectious causes including cytomegalovirus and clostridium difficile were ruled out; a rectosigmoidoscopy revealed diffuse superficial ulceration with pseudomembranes in the rectum and the sigmoid; upper limit of lesions was not seen. Biopsies showed architectural mucosal distortion with polymorph nuclear infiltrates and abscesses, compatible with active UC with no cytomegalovirus (CMV) inclusion. She was started on intravenous (IV) steroids as an induction therapy with marked clinical and biological improvement. Adalimumab infusion, subcutaneously, was initiated as a maintenance therapy at a dose of 160 mg followed by 80 mg after 2 weeks and then 40 mg every other week. Eight months later, a surveillance colonoscopy showed complete regression of the giant pseudopolyp (Figures 3 and 4) with partial endoscopic remission of the previously noted inflammation of rectum and sigmoid.

## 2. Discussion

We reported a new case of giant inflammatory polyp (GIP) with 2 major specifications: patient known to have left-sided UC and the GIP was localized in the caecum surrounded by normal macroscopic mucosa; the second characteristic is the complete repression of the GIP after introduction of biologic treatment.

GIPs are a rare finding in inflammatory bowel disease (IBD) patients. They appear after a prolonged periods of inflammation followed by healing processes [1–4] with predilection location in the transverse colon, followed by the sigmoid, descending colon, and cecum [3, 4]. GIP occurs in patients with UC, where disease extent and activity were documented; the majority (14/19, 70%) had extensive colitis and had quiescent disease (25/38, 66%) at the time of presentation [5]

Two untypical caecal GIP in IBD patients has been reported in the literature. The first one in a patient with distal UC and was attributed to a caecal patch or a pan colitis that had subsequently regressed [6]. The second case with known Crohn's disease in remission had a worm-like giant polyps of 13 cm in size growing up from the ileocecum surrounded by reddish and edematous mucosa and was treated surgically [4]. But, our patient with known left-sided UC had a GIP in the cecum surrounded by normal mucosa endoscopically.

Often, GIPs present as finger-like projections of the mucosa, the so-called filiform appearance. Polyps can form clusters and present as warm like projections [1]. However, our patient has an adenoma-like polyp with broad base and definite borders and smooth surface but without exudates, which is rare or unusual.

Differentiating benign giant pseudopolyps from dysplasia-associated lesion is ongoing clinical challenge. Only two cases of GIP associated carcinoma or high grade dysplasia in IBD patients are described in the literature; in other words, malignant transformation of a pseudopolyp is rare [1]. This transformation was reported to be related to the repetitive episodes of severe inflammation and the increased extent of colitis [7]. The risk of colorectal cancer is increased in patients with IBD and history of pseudopolyps or GIPs and is found to be 2-fold greater than the general population [8]; so frequent endoscopic follow-up is needed and suggested to be done at 3-year interval by United Kingdom guidelines, at 2- or 3-year intervals by the European Crohn's and Colitis Organization and between 1- and 3-year intervals by the American Society for Gastrointestinal Endoscopy [1].

Clinically, patients with GIP may have symptoms similar to IBD flare including anemia, weight loss, cramping abdominal pain, diarrhea, passage of blood through the rectum, and colonic obstruction. But most cases remain asymptomatic and go undiagnosed until they develop signs and symptoms of obstruction and hemorrhage [9]. Our patient was completely asymptomatic and the caecal GIP was discovered incidentally during colonoscopy for colorectal cancer screening.

However, as most patients present with obstructive symptoms, colectomy is usually the first approach (n=66/78, 85%), due to failure of conservative management or, more commonly, to exclude underlying malignancy [5]. Conservative management of GIP is possible, with endoscopic resection and subsequent surveillance [10]. Yong Sung Choi et al. reported two unusual cases of medically treated GIPs with partial regression: the first was a case of Crohn's disease with GIP in the sigmoid colon partially regressed after six months of steroids and azathioprine treatment; the second one was a recto sigmoid GIP in a patient with left-sided UC with gradual regression after 3 years of oral and local mesalamine therapy [7]. Our patient is the first case that showed a complete resolution of the caecal GIP within 8 months on adalimumab therapy.

## 3. Conclusion

Giant PIPs should be considered in the differential diagnosis in a patient with a colorectal mass lesion in the context of IBD. They are seen on an inflamed mucosa after a chronic process of inflammation and healing. Surgery is often the first approach since almost all patients presented with obstructing signs and symptoms. This report illustrates an unusual case of GIP on a normal colonic mucosa during a colonoscopy for colorectal cancer screening in an asymptomatic patient with left-sided ulcerative colitis, showing complete resolution after 8 months of adalimumab therapy.
